# A Learning Health System Approach to Developing a Perinatal Safety Framework and Guide to Reduce Disparities in Maternal Harm

**DOI:** 10.1002/lrh2.70057

**Published:** 2025-12-05

**Authors:** Angela D. Thomas, Tamika Auguste, Allan Fong, Aaron Z. Hettinger, Seth Krevat, Laura Lee, Emily Mutondo, Deborah F. Perry, Karey Sutton, Saanvi Garg, Loral Patchen

**Affiliations:** ^1^ MedStar Health Columbia Maryland USA; ^2^ MedStar Health Research Institute Columbia Maryland USA; ^3^ Georgetown University Washington DC USA; ^4^ MedStar Washington Hospital Center Washington DC USA; ^5^ MedStar Health Institute for Quality and Safety Columbia Maryland USA

**Keywords:** health equity, learning health system, maternal disparities, maternal safety

## Abstract

**Introduction:**

Maternal harm disproportionately affects Black birthing individuals, with systemic and provider‐related factors contributing significantly to preventable severe maternal morbidity (SMM) and maternal mortality. Despite the severity of the crisis, traditional maternal safety approaches, which typically exclude patient voices, narrowly focus on severe harm events rather than conducting full‐spectrum safety surveillance, which limits proactive intervention strategies.

**Methods:**

This study applied the National Academies of Medicine's Learning Health System (LHS) principles to develop a comprehensive maternal safety framework. We employed a mixed‐methods approach integrating patient‐reported experiences of cases (patients experiencing severe maternal morbidity and/or postpartum readmission) and controls, provider perspectives, clinical informatics, natural language processing of clinical notes, and chart reviews to identify factors contributing to and mitigating maternal harm. We convened an interdisciplinary expert panel to synthesize findings into actionable recommendations for a Perinatal Safety Framework and a Perinatal Strategy Guide.

**Results:**

The Perinatal Maternal Safety Framework was developed to define the maternal safety continuum. Six key features emerged, including: (1) three distinct starting points (pregnancy, birth, postpartum), (2) variability in baseline circumstances, (3) a broad definition of maternal harm encompassing physical, emotional, and psychological factors, (4) adaptation of established safety models, (5) six categories of contributing and mitigating factors, and (6) status factors influencing maternal safety outcomes. The Perinatal Strategy Guide outlines evidence‐based strategies addressing structural and systemic factors affecting maternal safety.

**Conclusions:**

A comprehensive maternal safety framework integrating full‐spectrum safety surveillance and patient‐centered reporting is critical to addressing maternal health disparities. By applying LHS principles, this study provides a data‐driven, equity‐focused approach to improving maternal safety. Implementation of the Perinatal Strategy Guide will require interdisciplinary collaboration and engagement of individuals with lived experience to drive systemic change.

## 1 Introduction

Maternal harm is a major crisis disproportionately affecting Black birthing individuals [1]. In a multiyear national study, the Centers for Disease Control and Prevention found that Black women were three times more likely to experience maternal harm than White women—four to five times for women over age 30 [2]. The vast majority of severe maternal morbidity events (nearly 90%) and most maternal deaths (approximately 60%) are preventable [2, 3], with the most frequent preventable factors found to be provider‐related and/or system‐related, including substandard providers, delay or failure to diagnose or recognize high‐risk status, and delayed or inappropriate treatment [4]. Given that these provider‐ and system‐related contributing factors disproportionately affect historically marginalized groups [5], this preventable maternal harm crisis is better defined as a preventable maternal patient safety crisis that disproportionately affects Black birthing individuals.

**FIGURE 1:**
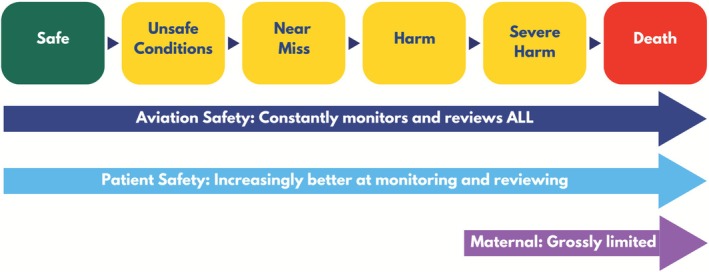
Comparing the spectrum of safety surveillance for aviation safety, patient safety, and maternal safety.

Despite the severity of the crisis, maternal safety has lagged behind other safety disciplines, like aviation safety and other patient safety areas, by narrowly focusing on severe maternal morbidity and maternal mortality (Figure 1). This is the equivalent of aviation safety experts only investigating plane crashes that are fatal, or non‐fatal but cause severe injury. Aviation safety experts conduct regular surveillance across a full spectrum of safety to uncover unsafe conditions, hazards, near misses, and other injuries that do not result in a crash [6]. Once uncovered, aviation safety experts use these incidents to identify common themes and signals to develop interventions along the full aviation safety spectrum, to prevent crashes before they occur [6]. Some medical specialties model patient safety after aviation by investigating patient harm events resulting in serious injury or death and conducting surveillance across a full spectrum of patient safety to prevent patient injury and death [7].

Given the magnitude and disparity of the maternal harm crisis, maternal safety must align with the National Academies of Medicine's Learning Health System (LHS) principles [8]. These principles emphasize a patient‐centered, evidence‐based system that is continuously learning and improving [8]. Maternal safety must model its practices after aviation safety and patient safety by conducting surveillance across a full spectrum of preventable maternal harm. However, traditional patient safety reporting surveillance alone will not address the disproportionate harm that Black birthing individuals face, as those approaches often miss those individuals experiences of mistreatment by relying on administrative coding and reports by healthcare staff. Our research shows that in voluntary occurrence reporting systems, healthcare staff are significantly more likely to report harm and near miss events when the patient is White versus when the patient is Black [9, 10]. This differential reporting by a patient's race leads to disparities in learning from patient safety events and potentially contributes to disparities in harm such as those highlighted in The Giving Voice to Mothers Study, which uncovered experiences of racism, discrimination, and mistreatment during the perinatal period [11]. Thus, maternal patient safety reporting also needs updated equity‐informed approaches to uncover factors that contribute to maternal harm.

**FIGURE 2:**
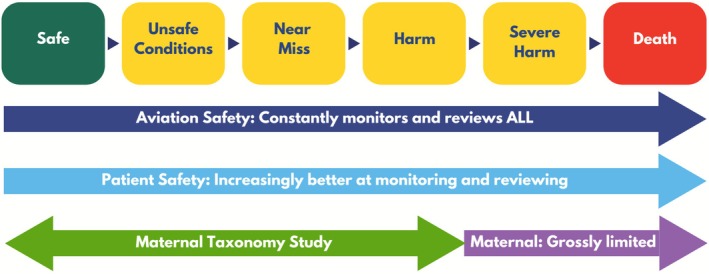
The maternal taxonomy study addresses the full spectrum of safety surveillance for maternal safety to align with aviation safety and patient safety surveillance.

In alignment with the LHS principles of inclusivity and equity, elevating the voices of birthing individuals, especially those from marginalized communities, is essential for bridging gaps in understanding maternal safety. Despite awareness of the preventable maternal safety crisis disproportionately affecting Black birthing individuals [11, 12, 13, 14, 15, 16, 17, 18, 19, 20], maternal safety approaches have lacked a full spectrum understanding of harm. Without full spectrum safety surveillance, there has been no way to further understand what leads to the most severe upstream outcomes for birthing individuals, let alone prevent those outcomes. What is needed is a comprehensive, continuously learning maternal safety framework that integrates patient experiences to fully account for racism, discrimination, and implicit bias across the full spectrum of maternal harm (Figure 2).

The voices of birthing individuals are critical for understanding upstream factors that contribute to unsafe conditions, near misses, and maternal mortality, revealing insights that traditional reporting often overlooks. Integrating other signals such as electronic health record (EHR) data, provider and staff insights, and chart abstraction aligns with the LHS principle of leveraging data and analytics to drive learning and improvement [8]. This multidimensional approach supports the development of targeted interventions to address and mitigate risks effectively.

The purpose of this study was to use LHS principles to develop a comprehensive maternal safety framework that illustrates the progression from a safe birth to conditions that become unsafe, transition from unsafe to near misses, and ultimately result in severe maternal harm or mortality. This framework reflects key themes that contribute to maternal harm and those that can mitigate it. Using the key themes identified, an interdisciplinary expert panel developed actionable recommendations for improving maternal care in a perinatal safety guide. The perinatal safety guide is designed for any clinical or operational leader who is responsible for ensuring safe maternal healthcare delivery. The leaders can implement the strategies in this guide using a collaborative, interdisciplinary approach that optimally engages individuals from the community with lived experience as collaborators. By understanding the maternal safety continuum, healthcare leaders and clinical teams can better implement these strategies that promote safe maternal healthcare throughout the perinatal journey, thereby fostering a learning health system that is patient‐centered and equitable. The urgency of maternal and infant mortality in the United States, particularly among Black women, underscores the need for such frameworks and guides to address structural determinants of health and longstanding inequities in care.

## 2 Methods

This study aimed to inform the development of the Perinatal Maternal Safety Framework and the Perinatal Strategy Guide to address preventable maternal harm throughout the perinatal period. Guided by the National Academies of Medicine's Learning Health System (LHS) principles—including person‐centeredness, equity, data‐driven learning, and continuous improvement—this study employed a multifaceted methodology to capture diverse insights into maternal safety [8]. We integrated patient voices, clinical data, and provider perspectives to ensure a comprehensive understanding of preventable maternal harm. We developed a strategy guide that describes key themes and recommended strategies to support healthcare organizations efforts to ensure maternal safety throughout the perinatal journey.

### 2.1 Study Design and Participant Recruitment

We used a case–control model to identify factors contributing to maternal harm. Cases included birthing individuals who experienced severe maternal morbidity (SMM) events, as defined by the Centers for Disease Control and Prevention's 21 indicators [21], and/or postpartum readmission within 42 days of delivery. Controls were birthing individuals matched by age, race, and insurance status who did not experience SMM events or postpartum readmission and delivered a healthy, term infant (≥ 37 weeks, ≥ 2500 g) without the use of vacuum or forceps assistance. Individuals involved in risk management claims were excluded to avoid causing additional distress to birthing individuals caused by revisiting adverse experiences and to minimize institutional legal risk.

### 2.2 Surveys and Interviews

#### 2.2.1 Online Birthing Individual Survey

We invited 1426 eligible birthing individuals who met the case–control criteria described above and received care between 2017 and 2019 at one of two large urban hospitals, one a high‐volume tertiary care center and the other an academic medical center affiliated with a major university, to participate in an online survey about their perinatal experiences. The survey adapted instruments used in the Giving Voice to Mothers Study: Mistreatment by Care Providers in Childbirth Indicators (MCPC Indicators), Mothers on Respect Index (MOR), and the Mothers Autonomy in Decision Making Scale (MADM) [11, 22, 23]. Collectively, these instruments assess communication breakdowns, bias, discrimination, and overall quality of maternal care. The survey also included two free‐text questions where respondents could share additional details about their experience overall and their perceptions of safety throughout the process. Respondents received $25 for their time.

#### 2.2.2 Virtual Interviews With Birthing Individuals

Of those invited, 284 individuals (20%) responded. We asked participants, both cases and controls from both hospitals, whose scores on the instruments reflected disrespect, mistreatment, and lack of autonomy in decision making to participate in a 1‐h qualitative, virtual interview to share additional details about their perinatal experiences. The virtual interviews included questions about birthing individuals' experiences during pregnancy, childbirth, and the postpartum period. We asked about care team interactions, support, concerns, and delays care. We also asked birthing individuals about their birth plans, communication with providers, and sources of stress or fear. We explored additional questions about satisfaction with care and advice for future birthing individuals. Respondents received $50 for their time. We qualitatively analyzed these interviews and identified recurring themes in maternal safety and care quality.

#### 2.2.3 Online Provider Survey

We invited the maternal health providers—including obstetricians, midwives, and perinatal nurses at the high‐volume tertiary care hospital to complete an online survey to share their perspectives and experiences in delivering safe, maternal care. We adapted the interview guide used by Howell et al. [24] to survey format using a Likert scale to assess perceptions of the quality and safety of maternal care including the impact of midwives and doulas, availability of working equipment and supplies, provider‐to‐provider communication, staff training, implicit bias, staffing adequacy, and how adverse outcomes maternal outcomes are addressed.

### 2.3 Other Data Collection and Analyses

#### 2.3.1 Clinical Informatics Signal Detection

We used clinical informatics techniques to detect early “signals” of unsafe maternal care delivery in the electronic health record (EHR) of both hospitals. This included primarily structured data analysis of diagnoses, procedures, medications, and diagnostic testing. We applied pattern recognition techniques to clinical workflows and documentation to identify safety concerns, stratified findings by race, and validated signals through chart abstraction and voluntary occurrence reports. These methods were aligned with LHS principles to support proactive safety surveillance and equity‐focused interventions.

#### 2.3.2 Natural Language Processing (NLP) for Tone and Sentiment

We used natural language processing (NLP) to detect negative tone and sentiment in EHR clinical notes. We analyzed five types of clinical notes at both hospitals: history and physical notes, review of systems documentation, history of present illness, physical examination, and triage notes. We first extracted sentences that contained one or more target terms. We used 15 target terms: nonadherent, aggressive, agitated, angry, challenging, combative, noncompliant, confront, uncooperative, defensive, exaggerate, hysterical, unpleasant, refuse, and resist. We then used inter‐rater reliability to assess if the contextual use of these words were likely indicative of negative tone and sentiment. Our approach is similar to that presented by Sun et al. [25]. We compared findings by race and by case/control status.

#### 2.3.3 Chart Reviews for Adverse Safety Events

We conducted in‐depth chart reviews for all participants who completed a qualitative interview (*N* = 50) to assess the presence or absence of adverse safety events (i.e., preventable harm) in the chart. A nurse trained in patient safety conducted each review using both the patient's chart and their interview transcript. A physician with expertise in patient safety collaborated with the nurse to finalize the assessment.

#### 2.3.4 Patient Safety Event and Patient Complaint Analyses

We analyzed voluntary occurrence reports at both hospitals from healthcare staff and patient complaints. This approach allowed us to incorporate both provider and patient‐reported adverse safety events and concerns. We identified key patient experience themes, dissatisfaction, and instances of disrespect, while also examining differences by race in how patients' voices were represented in these reports [26].

### 2.4 Interdisciplinary Expert Panel With Community and Provider Engagement

Aligning with the LHS principles of continuous learning and improvement, we convened two interdisciplinary panel meetings. The first meeting provided critical feedback on early data collection, which informed additional data collection and new analytic approaches. The second meeting focused on synthesizing the findings from all data collection efforts, finalizing the Perinatal Safety Framework, and generating actionable recommendations for the Perinatal Strategy Guide. The interdisciplinary panel included experts in maternal qualitative survey research, health equity, obstetrics, midwifery, nursing, human factors, patient safety, data science, informatics, biostatistics, health policy, behavioral health, patients with lived experience, and community.

### 2.5 Developing the Perinatal Safety Framework

Using the feedback from the interdisciplinary expert panel, we identified common themes emerging across the data sources to triangulate the data from the patient surveys, qualitative interviews, clinical informatics analyses, NLP analyses, provider surveys, chart reviews, and patient safety reports. This triangulation helped to construct the first draft of the comprehensive Perinatal Safety Framework, which was presented to the interdisciplinary expert panel prior to the second meeting for additional input and feedback to refine and finalize the framework. Aligning with the LHS principles of equity and person‐centeredness, we prioritized the voices of birthing individuals, especially those from historically marginalized groups. For example, if during chart review, an experience of a birthing individual was not reflected in the chart (e.g., “my complaints about pain were ignored”) and that experience could have contributed to an adverse outcome, we included this information as a contributing factor in our analysis, even if it was not reflected in the electronic health record. We identified systemic issues, provider‐related contributing factors and mitigating factors to maternal harm. We also identified factors present upon entry into the care delivery experience that are favorable or unfavorable for perinatal safety. This framework offers evidence‐based, patient‐informed strategies to improve maternal safety and advance equitable, sustainable maternal care improvements.

## 3 Results

### 3.1 Perinatal Maternal Safety Framework

**FIGURE 3:**
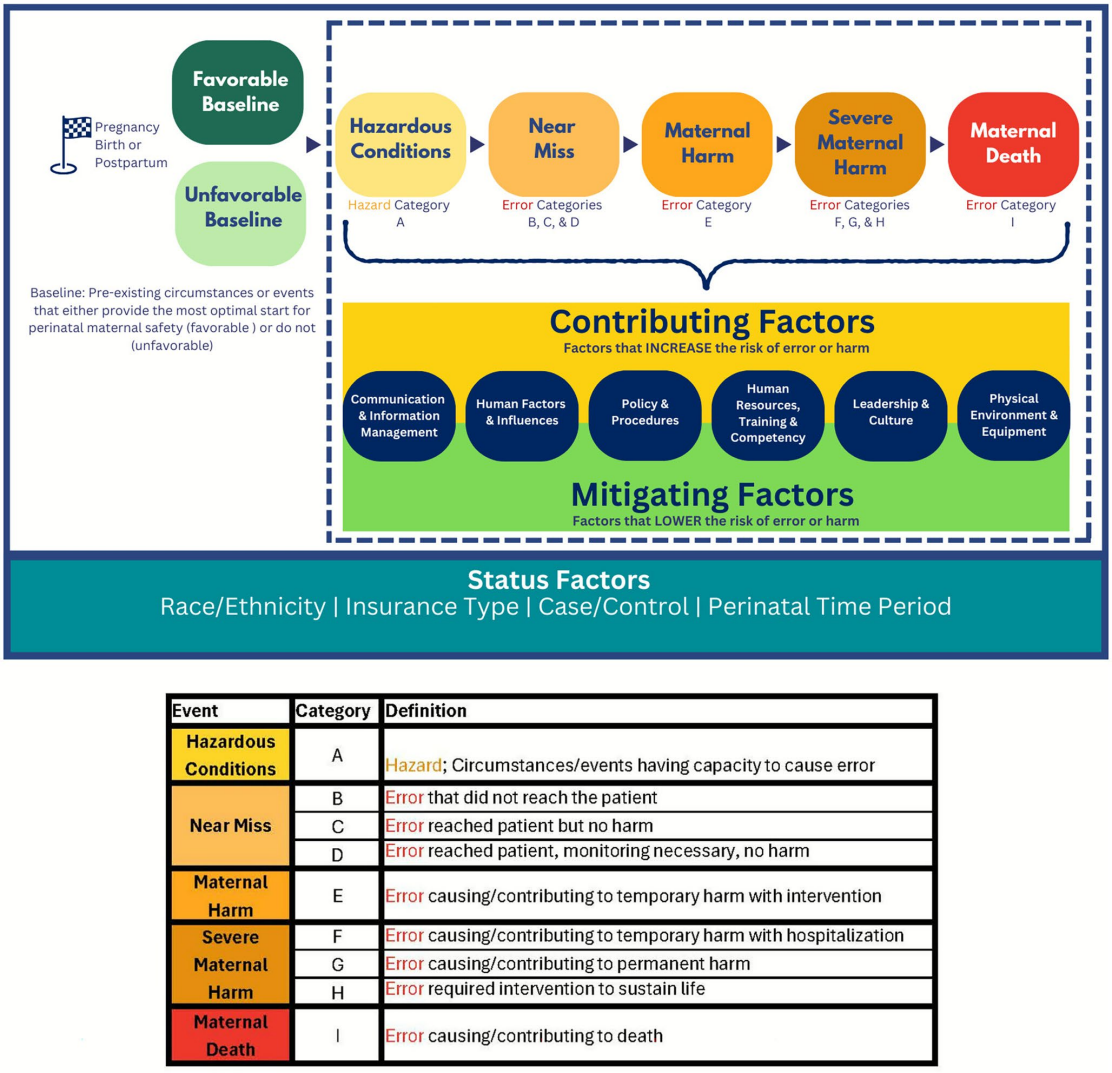
Perinatal safety framework.

Using an interdisciplinary analysis of all data collected, we developed the Perinatal Maternal Safety Framework (Figure 3) to comprehensively describe the maternal safety continuum throughout the perinatal period.

The Perinatal Safety Framework has six key features:

1. Three Distinct Starting Points in the Maternal Safety Journey: There are three separate starting points to the maternal safety journey: pregnancy, birth, and postpartum. This framing appreciates that a birthing person could have a safe, uneventful pregnancy, but an unsafe birth and likewise, a birthing person could have a safe, uneventful pregnancy and birth, but an unsafe postpartum period.
2. Variability in Baseline Circumstances: Every birthing person does not begin the maternal safety journey at the same baseline. Some begin pregnancy, birth or postpartum (or all three) with pre‐existing circumstances or events that do not provide the most optimal start for perinatal maternal safety.
3. Comprehensive Definition of Maternal Harm: Consistent with the National Coordinating Council for Medication Error Reporting and Prevention, the framework expands the definition of maternal harm to include physical, emotional, and psychological harm [27]. This holistic approach ensures that the full spectrum of maternal experiences is considered in safety improvement efforts.
4. Application of Established Safety Models: The framework adapts the National Coordinating Council for Medication Error Reporting and Prevention's index for categorizing medication errors to classify hazards and errors contributing to maternal harm [27]. This structured approach allows for systematic identification and analysis of safety risks.
5. Six Categories of Contributing and Mitigating Factors: We placed factors influencing maternal safety outcomes into six categories, reflecting risks that increase the likelihood of maternal harm “contributing factors” and practices that mitigate harm “mitigating factors”. Table 1 describes each category, including the equity lens we also applied in each category.
6. Status Factors Influencing Maternal Safety: We identified certain patient‐level attributes, referred to as “Status Factors” that influenced maternal safety outcomes. These included patient race/ethnicity, insurance type, and classification as either a case (severe maternal morbidity or postpartum readmission) or control (no severe maternal morbidity or postpartum readmission). The specific perinatal phase (pregnancy, birth, postpartum) also influenced risk. Recognizing the influence of these attributes is crucial for tailoring equitable care interventions.

**TABLE 1:** Contributing and mitigating factors.

Category	Description
Communication and information management	Availability of all necessary information, accuracy and completeness of information, adequacy of communication among staff, barriers to communication, hand‐offs, and transitions Equity Lens: language barriers, non‐verbal cues and style, literacy level, psychological safety, cultural norms and barriers, and modes of communication (e.g., portal, phone, text)
Human factors and influences	Cognitive biases (e.g., confirmation, anchoring, premature closure), distractions, usability, fatigue, stress, and patient factors Equity lens: stereotyping patient behavior, prejudice, implicit biases (e.g., based on socio‐economic status, race, ethnicity, language barrier, substance abuse, mental health, disability), EHR Design, and accessibility of resources
Policies and procedures	Presence of policies and procedures, clarity of policies to the end user, adherence with policies and procedures, and culture related to attention and adherence to policies. Equity lens: variable application of policies, clinical algorithms and medical devices using race as a biological construct, policies regarding collection of patient data (e.g., race, gender)
Human resources, training, and competency	Qualifications and competency of staff, staff training and orientation (e.g., job specific, ongoing, special training), supervision of staff, and staffing levels Equity lens: lack of training on implicit bias, equitable staffing levels, diversity of care team, and cultural humility modeling
Leadership and culture	Conduciveness of the culture to identifying risks and escalating information about adverse events (e.g., psychological safety and just culture), barriers to effective (non‐punitive) communication, and the influence of hierarchy, supervision, and organizational support Equity lens: This category includes attention to systemic barriers and biases, support and funding for health equity training, cultural humility modeling, and equitable allocation of resources
Physical environment and equipment	Availability, usability, location, codes, and specs of equipment/technology. Preventative maintenance, appropriateness, standardization, and environmental hazards in the physical environment Equity lens: Weight‐centric equipment, home health usability, equipment equity for all skin tones, and ADA‐compliant physical spaces

### 3.2 The Perinatal Strategy Guide for Maternal Safety: Summary of Key Findings and Themes

The Perinatal Strategy Guide builds upon the Perinatal Maternal Safety Framework by translating key findings into practical recommendations that align with the LHS principles of fostering continuous learning, data‐driven decision‐making, and system‐wide improvements. The following summary highlights the key findings and strategic themes identified by the interdisciplinary panel:

1. *Baseline Factors*: Disparities in behavioral health support and social support were most present during pregnancy and postpartum, contributing to adverse maternal safety outcomes; emphasizing the need for proactive, equity‐focused interventions to address behavioral health and social support needs.
2. *Contributing and Mitigating Factors*: Systemic barriers such as fragmented care continuity, ineffective communication, and limited access to culturally responsive care contributed significantly to maternal harm. Addressing these issues requires the implementation of evidence‐based protocols, adherence to those protocols, and seamless care coordination across disciplines to mitigate the risk of maternal harm.
3. *Status Factors*: Persistent disparities in maternal outcomes were linked to race, insurance type, and parity. Recognizing these status factors as risk factors is essential for designing interventions that promote equity and improve safety for all patient populations.

### 3.3 The Perinatal Strategy Guide for Maternal Safety: Recommended Strategies

The comprehensive table labeled “Recommended Strategies from The Perinatal Strategy Guide for Maternal Safety” (Table 2) outlines seven key categories of recommended strategies for improving maternal care:

1. *Baseline Factors*: Recommended strategies highlight the need for screening and addressing behavioral health, social support, and high‐risk clinical needs during pregnancy, birth, and postpartum through standardized processes and patient education to enhance maternal safety.
2. *Communication and Information Management*: Recommended strategies promote respectful maternal care and ensure patient autonomy. This includes patient surveys to track care quality, mechanisms for reporting unkind treatment, and detailed discussions on birth plans to align patient preferences with clinical decisions.
3. *Human Factors, Bias, and Influence*: Recommended strategies include leveraging EHR technology to identify discriminatory language and employing AI simulations to train providers on non‐biased decision‐making. Strategies also include the need for inclusive care algorithms and bias‐mitigation protocols.
4. *Policies and Procedures*: Recommended strategies emphasize combating implicit bias through training and review processes, allocating adequate time for patient‐provider communication, and integrating high‐risk care bundles for comprehensive maternal care.
5. *Human Resources, Training, and Competency*: Recommended strategies include ensuring proficiency training for learners and providers in maternal care procedures, such as epidural placement and pain management. Strategies also stress proper staffing levels and support the inclusion of formal health advocates.
6. *Leadership and Culture*: Recommended strategies emphasize prioritizing maternal safety and fostering a supportive environment for providers. The strategies also include ensuring maternal health data are visible and used to identify and reduce disparities.
7. *Physical Environment and Equipment*: Recommended strategies stress that facilities should be clean, welcoming, and equipped with tools suitable for diverse patient needs and the proper resources must be available to providers and staff to support safe, high‐quality maternal care delivery.

**TABLE 2:** Recommended strategies from the perinatal strategy guide for maternal safety.

Key themes	Recommended strategies
*Baseline*	
Behavioral health and social support screening and interventions	Organization has an established process to screen for and address behavioral health needs during pregnancy, at birth, and during postpartum Organization has an established process to screen for social support needs during pregnancy, at birth, and during postpartum
Process to identify and provide additional monitoring and support for high‐risk clinical needs during pregnancy, birth, and postpartum periods	Organization has an established process for informing with high‐risk clinical needs that educates the patient about the increased risk(s) and encourages self‐advocacy Organization has an established process for acknowledging, monitoring, and providing additional support for high‐risk clinical needs, including, but not limited to situations when: ○ Birth begins with a clinically indicated labor induction ○ Birth begins with meconium present at just under 41 weeks ○ Postpartum begins with surgery complications from birth ○ Characteristics about the patient's anatomy increases risk
*Communication and information management*	
Providers deliver kind and respectful maternal care	Organization collects, tracks, assesses, and evaluates measures of kind, respectful maternal care delivery on patient experience surveys Organization offers each patient at each care delivery visit to report unkind, disrespectful maternal care Organization offers providers and healthcare staff a mechanism to report unkind, disrespectful maternal care Organization has a team dedicated to addressing complaints of unkind and disrespectful maternal care
Providers ensure patient autonomy in decision‐making and informed care delivery	Organization offers group prenatal care Federal, local, and reimbursement polices support organizational efforts to ensure prenatal and postpartum appointment visit time allocated allows for thorough, unrushed, and informed patient‐provider communication Prior to childbirth, providers discuss patient's birth plan, including scenarios where rapid decision‐making is critical to manage patient expectations and understand patient preferences in advance Providers ask patients how involve they want to be in decision‐making Providers inform patients of different options for maternity care Providers explain the advantages and disadvantages of maternity care options Providers help patients understand all the information Providers give patients enough time to thoroughly consider the different care options Providers give patients the ability to choose what the patient considers to be the best care options Provider respects patients' choices
Organization promotes effective provider to patient communication	Maternal care is accessible and provided by a consistent care team (i.e., team knows the patient well and the patient knows the care team well) throughout the perinatal journey
Organization informs, educates, and empowers patients	Organization provides a variety of educational opportunities and resources throughout the perinatal journey that empower patients to know their rights and what to expect while navigating the system Organization reminds each patient at each care delivery of their right to advocate for themselves along with a structured mechanism to escalate concerns Organization incorporates technology solutions to inform, educate, and empower patients
Organizations ensure proper provider to provider communication	Organization has established, effective, and efficient processes and procedures to ensure proper provider handoffs Organization has established processes and procedures to evaluate the efficacy of provider handoffs Organization ensures adequacy of staffing of perinatal care providers Organization ensures adequate interdisciplinary support across perinatal care provider roles (e.g., physicians, nurses, midwives, anesthesiologists)
Organization policies, procedures, and culture support the inclusion of formal health supports	Organization policies, procedures, and culture support the inclusion of internal and external doulas in maternal care delivery settings Organization highlights and promotes the availability of patient advocates (if available in the organization)
*Human factors, bias, and influence*	
Organization ensures non‐biased, non‐ discriminatory maternal care delivery	Organization uses EHR surveillance technology to identify potentially biased and discriminatory language in clinical notes Organization leverages artificial intelligence to deliver simulations and roleplaying exercises designed to train providers in making non‐biased, non‐discriminatory care decisions Organization uses artificial intelligence in the electronic health record to signal potential issues in delivering high quality maternal care delivery Organization ensures that algorithms used to guide care are free from bias
*Policies and procedures*	
Organizations address explicit and implicit biases	Organizations establish and implement processes that ensure that events related to bias, maternal morbidity, and maternal mortality are reported and receive a thorough safety event review Organizations engage providers in the evaluation of implicit bias training Organization implements system‐based solutions that promote cognitive pauses that mitigate and combat implicit bias Organization flags potentially biased statements in clinical notes for review and modification until the statement is acceptable
Organization ensures adequate time for patients and provider communication	Federal, local, and reimbursement polices support organizational efforts to ensure scheduling processes allocate sufficient prenatal and postpartum appointment visit time for thorough, unrushed, and informed patient‐provider communication Organization supports efforts to change reimbursement practices and policies that promote longer visit times Organization incentivizes positive maternal health outcomes over patient volume
Providers deliver evidenced based care equitably (e.g., treatment for anemia, gestational hypertension, gestational diabetes)	Organization has established, documented, and EHR‐integrated high‐risk care bundles for diabetes, depression and anxiety, hypertension/hypertensive disorders of pregnancy, anemia, expectant adolescents, and HIV
Increased screening and interventions for high risk social and clinical factors	Providers universally screen for behavioral health needs and social determinants of health in at least three timepoints during the pregnancy, with additional screens at childbirth and postpartum Organization has a system and process for tracking and following up on missed appointments for high‐risk patients Organization incorporates social identity into care plans to mitigate inequities and reduce relative risk
Organization prevents delays in care	Organization allows and encourages patients during the first prenatal visit to proactively schedule all prenatal appointments from first visit through birth Organization offers a dyadic framework for postpartum care during pediatric visits Organization offers multiple patient touch points and follow‐ups in a variety of mechanisms (e.g., chatbots, apps, doulas, clinic visits) during the first 42 days postpartum emphasizing symptom management, connection with resources, and psychosocial and emotional wellbeing Organization has embedded flags for when a patient has current and/or a history of mild, moderate, or severe preeclampsia in the EHR
Organization prevents discharge errors	Organization requires strict criteria for being discharged that includes physical, mental, and emotional assessments of readiness
Organization monitors, tracks, addresses, and evaluates patient experiences throughout the perinatal journey	Organization continuously elevates the patient voice throughout the perinatal journey to assess patient experience, including autonomy in decision making and respectful maternal care delivery
Organization proactively monitors, tracks, addresses, and evaluates severe maternal outcomes	Organization has an established panel that includes an equity expert to review each case of severe maternal morbidity to assess preventability, opportunities to improve care delivery, action planning, and action plan evaluation
*Human resources, training, and competency*	
Provider and learner proficiency of epidural placement	Organization establishes an evidence‐based, equity‐based training and evaluation process for learners for epidural placement that ensures proficiency during care delivery Organization establishes a process for identifying and re‐training providers and learners to increase proficiency in epidural placement
Proper expectation setting with patients about learners and the learning environment (e.g., residents)	Organizations who include learners (e.g., residents) in the maternal care delivery process have a structured and patient‐centered process for informing patients throughout the perinatal journey about the involvement of learners in care delivery ○ This process should include the ability for patients to ask questions and express concerns
Learner competency for maternal care delivery	Organization establishes an evidence‐based, equity‐based training and evaluation process for learners for maternal care delivery that includes, but is not limited to: ○ Simulations of trauma informed care ○ Proper pain management during birth Organization establishes a process for identifying and re‐training providers and learners to increase proficiency
Proper staffing levels	Organization ensures proper staffing levels per nationally recognized guidelines for perinatal units, such as the Association of Women's Health, Obstetric and Neonatal’ Standards for Professional Registered Nurse Staffing for Perinatal Units
Availability of formal health supports	Organization policies, procedures, and culture support the inclusion of internal and external doulas in maternal care delivery settings Organization highlights and promotes the availability of patient advocates (if available in the organization)
*Leadership and culture*	
Providers and staff feel maternal health services are valued by the organization	Maternal safety is included as a strategic priority in annual and strategic plans at the highest levels of the organization Maternal safety is included as a strategic priority in annual and strategic plans at the local hospital Positive recognition of providers and staff delivering maternal health services from organizational and local leadership
Providers and staff have hope that maternal health disparities can be improved	Organization collects, tracks, assesses, and evaluates, maternal health disparities across clinical outcomes and patient experience Organization ensures visibility of maternal health disparity data to providers and healthcare staff Organization recognizes and celebrates reductions in maternal health disparities Facilities allow for high quality care of natural childbirth patients Facilities allow for high quality care of high‐risk patients
Organization promotes wellbeing for providers and staff providing maternal health services	Organization provides a psychologically safe environment that offers a safe space to speak up Organization provides an esthetically welcoming and physically safe workplace environment Organization provides the proper resources and equipment for providers and staff to deliver safe maternal health services Organization provides structured programs, procedures, and processes that mitigate the high‐stress provider work environment (time constraints, stress, patient load, etc.)
*Physical environment and equipment*	
Maternal health facilities that are welcoming and inviting for optimal patient and provider experience	Organization's perinatal care facilities allow for high quality care of patients choosing natural childbirth Organization's perinatal care facilities allow for high quality care of high‐risk patients Organization provides an esthetically welcoming, inviting, clean and sanitary maternal care environment
Proper equipment for high quality maternal care delivery is present, available, and in working order	Organization provides the proper resources and equipment for providers and staff to deliver safe, equitable, high quality maternal health services Organization ensures available equipment is suitable to properly assess, evaluate, diagnose, and treat all patients regardless of size, skin tone, and any other personal characteristic

## 4 Discussion

This study developed a comprehensive maternal safety framework to understand the continuum from safe births to near‐miss events and, ultimately, maternal mortality. By integrating diverse data sources, including birthing individuals' voices, patient safety data, clinical informatics, clinical notes, provider voices, and chart abstractions, this framework enabled the identification of key themes contributing to maternal harm. The framework also informed targeted strategies to improve maternal care.

A major strength of this study lies in its incorporation of learning health system principles, centering the patient voice, fostering continuous improvement through comprehensive data analysis, and multi‐stakeholder engagement. Elevating the lived experiences of birthing individuals provided invaluable insights often underrepresented in patient safety efforts. The diverse interdisciplinary expert panel, consisting of professionals in maternal health, health equity, patient safety, human factors, and data science, enriched the analysis and the development of actionable recommendations. These recommendations, which are detailed in the strategy guide, align with approaches seen in the Safe Babies Safe Moms initiative, where interdisciplinary collaboration between providers, operational leaders, and community‐based organizations led to improved maternal and infant outcomes and reduced disparities [28]. Likewise, implementing the Perinatal Safety Framework and the Perinatal Safety Guide will require a collaborative, interdisciplinary approach.

Clinical and operational leaders responsible for ensuring safe maternal care delivery should assume responsibility for overseeing the implementation of the recommended strategies while engaging a dedicated team of other key clinical and operational leaders. This team will play a pivotal role in guiding and coordinating the execution of the strategies. Furthermore, these leaders should engage with other stakeholders relevant to implementing the specific strategies. Importantly, leaders must include individuals with lived experience in the community as collaborators to ensure birthing individuals represent their perspectives and needs throughout the process.

Despite these strengths, we must acknowledge several limitations. First, the patient survey data may be subject to recall bias, potentially affecting the accuracy of reported experiences. Because our analysis prioritized the perspectives of birthing individuals, any inaccuracies in their recollections may have been emphasized, potentially obscuring other important contributing factors. Second, the survey had a 20% response rate, which may introduce selection bias. Respondents may have been more motivated to participate due to particularly positive or negative experiences, potentially limiting the generalizability of the findings. Third, the exclusion of individuals involved in risk management claims may have led to the omission of cases with unique or more severe adverse experiences, potentially introducing bias into the study findings. Fourth, the retrospective nature of the clinical informatics data collection, chart abstraction, and patient safety system analyses limits the ability to infer causality. Fifth, the provider survey had a small sample size of only 26 respondents, which may not capture the full range of provider experiences. Sixth, the study was conducted within two hospitals from the same health system and geographic region, limiting generalizability to broader populations. Lastly, the data reflect pre‐pandemic births, and maternal care experiences may have evolved since then.

Despite these limitations, this work provides significant value to maternal safety initiatives. Our comprehensive approach and inclusion of the voices of birthing individuals highlight critical upstream factors that affect maternal outcomes. The diverse expertise involved in developing the strategy guide ensured well‐rounded, practical recommendations for healthcare providers. The Perinatal Safety Framework serves as a foundational tool for advancing maternal safety, particularly addressing disparities disproportionately affecting Black communities. Future research should expand on these findings by incorporating national patient and provider perspectives and identifying existing best practices to enhance the framework's applicability.

We have begun to catalyze learning from our findings and recommendations. We are actively partnering with a national organization to disseminate the framework and guide to maternal health, patient safety, and health equity audiences through diverse channels, including professional conferences, webinars, media outreach, social media, and collaboration with community influencers. Through the Safe Babies Safe Moms initiative, we have successfully implemented many of the Perinatal Safety Guide recommendations and are actively working with clinical and operational leaders to advance additional strategies [28]. Using the findings from our clinical informatics and natural language processing work, we are developing an EHR‐embedded surveillance tool that enables a timely feedback loop that flags missed maternal care delivery and negative tone or sentiment, supporting earlier intervention and ensuring that every birthing individual receives equitable, unbiased care [29]. By combining evaluation of current initiatives, future research, and feedback from collaborating organizations, we will refine and update the framework and guide to remain responsive to the changing needs of maternal health care.

To ensure successful implementation, healthcare organizations should identify a dedicated team of clinical and operational leaders responsible for executing the recommended strategies. By engaging, other key stakeholders as needed, organizations can determine the most effective ways to implement the strategies in the Perinatal Safety Guide. By adopting these insights and strategies, healthcare providers can make meaningful progress toward improving maternal safety and advancing equity in maternal care.

## Conflicts of Interest

The authors declare no conflicts of interest.

## Data Availability

The data that support the findings of this study are available on request from the corresponding author. The data are not publicly available due to privacy or ethical restrictions.
